# Consumption of Sugar-Sweetened Beverages, Juice, Artificially-Sweetened Soda and Bottled Water: An Australian Population Study

**DOI:** 10.3390/nu12030817

**Published:** 2020-03-19

**Authors:** Caroline Miller, Kerry Ettridge, Melanie Wakefield, Simone Pettigrew, John Coveney, David Roder, Sarah Durkin, Gary Wittert, Jane Martin, Joanne Dono

**Affiliations:** 1 School of Public Health, The University of Adelaide, Adelaide 5000, Australia; 2 Health Policy Centre, South Australian Health and Medical Research Institute, Adelaide 5000, Australia; Kerry.ettridge@sahmri.com (K.E.); Jo.dono@sahmri.com (J.D.); 3 Centre for Behavioural Research in Cancer, Cancer Council Victoria, Melbourne, Australia, 3004 & School of Psychological Sciences, The University of Melbourne, Melbourne 3010, Australia; melanie.wakefield@cancervic.org.au (M.W.); sarah.durkin@cancervic.org.au (S.D.); 4 Food Policy, The George Institute for Global Health, Sydney 2042, Australia; SPettigrew@georgeinstitute.org.au; 5 College of Nursing and Health Sciences, Flinders University, Adelaide 5042, Australia; john.coveney@flinders.edu.au; 6 Cancer Epidemiology and Population Health, University of South Australia, Adelaide 5000, Australia; david.roder@unisa.edu.au; 7 Discipline of Medicine, University of Adelaide, Adelaide, Australia, 5000 & Centre for Nutrition and GI Diseases, South Australian Health and Medical Research Institute, Adelaide 5000, Australia; gary.wittert@adelaide.edu.au; 8 Obesity Policy Coalition and Alcohol and Obesity Policy, Cancer Council Victoria, Melbourne 3004, Australia; jane.martin@cancervic.org.au

**Keywords:** sugar-sweetened beverages, fruit juice, artificially-sweetened beverages, consumption, national survey

## Abstract

Reducing consumption of free sugars, such as those found in high concentrations in manufactured products such as sugar-sweetened beverages (SSBs) and 100% fruit juices, is a global public health priority. This study aimed to measure prevalence of widely available pre-packaged non-alcoholic water-based beverages (carbonated sodas, sports drinks, energy drinks, artificially-sweetened sodas, fruit juices (any type), and bottled water) and to comprehensively examine behavioral, environmental, current health, and demographic correlates of consumption. A cross-sectional, nationally-representative population survey of 3430 Australian adults (18+ years) was conducted using computer-assisted telephone (mobile and landline) interviewing. Past week prevalence of pre-packaged drinks containing free sugar was 47.3%; daily prevalence was 13.6%. Of all the pre-packaged drinks assessed, consumption of fruit juices (any type) was the most prevalent (38.8%), followed by bottled water (37.4%), soda (28.9%), artificially-sweetened soda (18.1%), sports drinks (8.1%), and energy drinks (4.2%). Higher soda consumption was associated with males, younger age, socio-economic disadvantage, frequent takeaway food consumption, availability of soda in the home, obesity, and a diagnosis of heart disease or depression. A diagnosis of Type 2 Diabetes was associated with increased likelihood of consuming artificially-sweetened sodas and decreased likelihood of consuming sugar-sweetened soda. SSB consumption is prevalent in Australia, especially among young adults and males, foreshadowing continued population weight gain and high burdens of chronic disease. To reduce consumption, Australia must take a comprehensive approach, incorporating policy reform, effective community education, and active promotion of water.

## 1. Background

The World Health Organization recommends reducing the intake of ‘free sugars’ (i.e., “monosaccharides and disaccharides added foods and beverages by the manufacturer, cook or consumer, and sugars naturally present in honey, syrups, fruit juices and fruit juice concentrates”; p1, [1]) to less than 10% of total energy intake [1]. Sugar Sweetened Beverages (SSBs), defined as any consumable non-alcoholic water-based beverage that has sugar added to it, and 100% fruit juices contain significant amounts of free sugars. The World Health Organization guidelines state that consuming these products increases overall energy intake and may displace healthier foods from the diet, potentially leading to weight gain and increased risk of non-communicable disease [1]. Classifying 100% fruit juice as a drink that should be limited is not without controversy due to inconclusive evidence from multiple meta-analyses on the causal link between 100% fruit juice consumption and adverse health effects [2,3,4,5]. Nevertheless, 100% fruit juice is causally linked to tooth decay [6] and can contribute to excess calories to the diet [1], suggesting that 100% fruit juice is not an appropriate substitute for SSBs.

The strengthening evidence that SSB consumption is causally associated with increased risk of developing health problems, such as weight gain and obesity [7,8,9], Type 2 Diabetes Mellitus (T2DM) [10,11], tooth decay [12,13], and cardiovascular disease [14,15], coupled with rapid increases and widespread high consumption of SSBs, has prompted calls to action by governments from global public health organizations [1]. Numerous countries now tax SSBs with emerging evidence of reductions in population consumption [16,17,18,19,20]. Chile has gone further, leading globally with beverage warning labels [21], as part of a comprehensive suite of food and beverage regulation [22]. In Australia, like many countries, policy action is slow and legislative reform is conspicuously absent. This is despite strong and increasing consumer support for intervention [23], and comprehensive evidence-based guidelines for action [24].

Current systems designed to guide consumers’ dietary choices have limitations. While Australia’s Dietary Guidelines recommend consumers “limit” SSBs, they do not offer tangible quantification to help consumers understand what constitutes excess consumption [25]. Furthermore, Australia’s guidelines allow for 125 mL (approx. 4 fl oz) of juice to replace a serve of fruit “occasionally.” Australia and New Zealand have adopted an interpretive front-of-pack labelling scheme (Health Star Ratings); however, use of the scheme by manufacturers is voluntary and as a result there is low uptake across SSBs [26]. Furthermore, the scheme awards many fruit juices the healthiest ranking along with water. While social marketing campaigns targeting SSB consumption have been run in only two of eight Australian states and territories, they have demonstrated the ability to influence beverage consumption practices in those jurisdictions [27].

The lack of policy action is concerning in the context of very high rates of overweight and obesity 63% [28] increasing the preventable burden of non-communicable diseases [29], and widespread SSB consumption [30]. SSBs contribute 50% of Australians’ free sugar intake [31]. As a nation, Australia exhibits high rates of SSB consumption, ranking 15th in sales (kcal/cap/day) globally [32]. In addition, the Australasia region (i.e., Australia and New Zealand) has the second highest artificially-sweetened beverage sales, which are almost double the amount of all other regions apart from North America. Trends in SSB sales in Australia are consistent with worldwide trends, whereby sales of caloric soda have remained relatively stable, while sales of sports and energy drinks have increased. Sales of artificially-sweetened beverages are also reportedly increasing in Australia and elsewhere [30,32,33]. Effective public health responses to excessive free sugar consumption, and SSB consumption in particular, require an understanding of overall and beverage-specific patterns of consumption. To date, most studies on consumption trends have combined various drink types into an overall SSB measure, thereby preventing the identification of diverging trends between categories.

Advantages of conducting national population surveys include identifying correlates of consumption to provide a more nuanced picture of behaviors to enable targeting of interventions to where they are most needed. In Australia and internationally, males, younger adults, and the socio-economically disadvantaged are consistently reported as having higher SSB consumption rates [33,34,35,36,37,38,39,40,41]. The few studies that have explored correlates of specific drink types (e.g., sports drinks, energy drinks, fruit juice) have used inconsistent consumption definitions, resulting in non-comparable results, although the finding that males and younger people are more likely to be consumers has been consistently reported [34,39,40,41,42]. Results have been mixed for artificially-sweetened beverages, except for the consistent findings that consumption is more likely among females [35,36,39,43], and those with higher Body Mass Index (BMI) [39,43]. A small number of studies have explored lifestyle factors associated with SSB consumption [43,44,45] and sports and energy drinks in particular [42]; however, none were conducted with nationally representative samples. Nevertheless, these studies indicate that lifestyle factors such as physical activity and food-related behaviors (e.g., frequent fast food consumption) can have differential relationships with the consumption of various drink types, and warrant exploring in a nationally representative sample.

The aim of this study was to estimate the population prevalence of weekly consumption of widely available pre-packaged non-alcoholic water-based drinks (i.e., sold in most stores where beverages can be purchased), including those high in free sugar (i.e., soda, sports drinks, energy drinks, and fruit juices (any type)) and those that contain no sugar (i.e., artificially-sweetened soda, bottled water). Additional aims were to explore clustering of consumption of multiple drinks types regardless of sugar content and to explore simultaneously the socio-demographic, health, lifestyle, and environmental correlates of consumption for each drink type.

## 2. Methods

We conducted a national survey of Australians aged 18 years and older using computer-assisted telephone interviewing. The survey was conducted between February and April 2017 via random digit dialing of landline and mobile phones with a 35:65 split, in line with Australian’s telecommunication use [46]. Screening questions identified in-scope persons (i.e., aged 18 years and older, able to speak English) and where more than one in-scope person was present, the youngest male, followed by the youngest female, was selected to increase the proportion of younger respondents. An upper limit of six (mobile) or eight (landline) calls was made to each telephone number to achieve an interview, with interviews lasting an average of 21 min. The survey was pilot tested in January 2017 and minor adjustments were made to improve the flow and clarity of the questions. The study was approved by the University of Adelaide’s Human Research Ethics Committee (Approval code:H-2016-285), and participants provided verbal informed consent before commencing the survey.

### 2.1. Measures-Outcome Variables

#### 2.1.1. SSB Consumption in Past Week

Beverage consumption was derived from measures used in previous studies [47,48,49,50]. A SSB definition was provided to participants at the start of the survey: ‘Sugary drinks are soft drinks [soda] like Coke and lemonade, energy drinks like Red Bull, sports drinks like Gatorade, fruit flavored drinks like Schweppes mineral waters and mixers, all types of fruit juice and cordial, and excludes artificially sweetened drinks and those mixed with alcohol.’ Participants were asked: ‘During the past 7 days, on how many days did you drink at least one 250 mL cup of a sugary drink?’ and ‘On days when you do drink sugary drinks, how many cups per day do you usually have?’ Responses to these questions were multiplied to give an average amount consumed in the past week. Categories were formed as follows: none, moderate (consumed 1 to 6 cups) and high (consumed 7 or more cups) past-week consumption. Participants were also asked to report whether this amount was the same, more, or less than they would typically consume in a week.

#### 2.1.2. Consumption of Specific Drink Types

Participants were asked how often (either daily, weekly, monthly, less than monthly, or never) they usually drank a cup of each of the following drink types that are high in free sugar: soda, energy drinks, sports drinks, and fruit juice. (Note: participants were not asked to report separately on 100% fruit juice and fruit juice drinks.) They were also asked to report frequency of consumption of drink types containing no sugar: artificially-sweetened soda (such as Coke Zero) and pre-packaged bottled water. Responses were collapsed into two categories: ‘daily/weekly’ and ‘monthly or less’ for logistic regression analyses.

### 2.2. Measures–Predictor Variables

Demographic characteristics collected were: gender, age, parental status (parent or carer of at least one child under 18 years of age who lives in the same household at least some of the time), education (highest qualification), employment status, grocery buyer status, and whether they speak English at home. Postcode-derived area-level disadvantage [51] was also collected and assessed according to the Australian Bureau of Statistics’ Socio-Economic Indexes for Areas index of relative socio-economic disadvantage. This index uses postcodes to categorize areas based on a range of socio-economic factors such as employment status, income, and household status. Scores were also categorized into deciles that rank areas from the most disadvantaged (low score) to least disadvantaged (high score). In this study, deciles were further categorized into three groups to enable comparison between participants from the most (deciles 1–3), moderate (deciles 4–7), and least disadvantaged areas (deciles 8–10). Postcode-derived remoteness [52] was assessed according to the Australian Statistical Geography Standard, which organizes discrete parcels of land in a hierarchy from ‘metropolitan’ to ‘very remote’ based on relative access to services. In this study, three mutually exclusive categories of remoteness were used: ‘major cities,’ ‘inner regional,’ and ‘outer regional, remote or very remote.’ Current health measures were: BMI (from self-reported height and weight); weight perception (assessed by asking participants whether they considered themselves to be an acceptable weight, underweight, or overweight; [53]); and health diagnoses (whether respondents had ever been told by a doctor or health professional that they had any of the following health conditions: T2DM, heart disease, arthritis or gout, depression, or a lung condition such as asthma or COPD (Chronic Obstructive Pulmonary Disease)).

Behavioral measures included: takeaway food consumption in the past 7 days (not at all, 1–2 times, 3 or more times); number of physically active days in the past week (none, 1–6 days, 7 days); average number of hours of sleep per night (6 or less, 7 to 8, 9 or more); participation in shift work (yes/no); consumption of alcohol and spirit-based alcoholic drinks that are mixed with sugary drinks (‘daily/weekly’ and ‘monthly or less’).

Environmental measures were: availability in the home (never, seldom/sometimes, almost always/always) of: soda, artificially-sweetened soda, and fruit juice.

### 2.3. Analyses

Chi-square tests of association between consumption and demographic characteristics were undertaken for each drink type. Two logistic regressions were carried out for SSB consumption in the past week using (1) none versus any consumption and (2) ‘moderate’ (1 to 6 cups) versus ‘high’ (7 or more cups) consumption as the outcome variables, respectively. A stepped process was used, whereby the first step tested demographic characteristics only and the second step tested current health and behavioral and environmental factors simultaneously, while controlling for the first step. Separate logistic regressions were also conducted for each of the following drink types: soda, energy drinks, sports drinks, fruit juice, artificially-sweetened soda, and pre-packed bottled water. For this, a stepped process was used, whereby step one tested demographic characteristics only, step two tested the consumption of other drink types, and step three tested current health and behavioral and environmental factors simultaneously, controlling for the previous steps. Each logistic regression analysis was repeated for males and females separately to assess whether the patterns of association differed according to gender.

A two-stage weighting procedure was used. First, a design weight was applied that adjusted for the individual’s chance of selection. Second, a post-stratification weight was applied to ensure the final sample was weighted to relevant population benchmarks for age, gender, location, and telephony status (landline vs. mobile). The weighting was applied for all of the results reported in this paper. A conservative cut-off value of *p* < 0.01 was used for reporting statistical significance.

## 3. Results

In total, 3430 participants were recruited into the study (participation rate = 44%). The sample is described in detail and compared to national population data elsewhere [23]. Briefly, 49% were female, mean age was 46.9 (Standard Deviation [SD] = 18.3) years, and the demographic profile (in terms of sex, age, and employment status) of respondents was consistent with the national population data [54]. The sample had slightly lower levels of overweight/obesity (53%) than found in a 2017/2018 national data set (67%) [30], likely due to different methods of assessment used between studies (self-report in this study vs. biometric assessment for the comparative data set).

Table 1 displays prevalence of consumption for each drink type. Almost half (47.3%) of participants had consumed an SSB in the past week, with 13.6% having at least one drink a day on average. More than half of participants reported they consumed soda (63.6%) or fruit juices (any type) (67.8%) at least occasionally. The proportion of participants reporting that they consumed sports and energy drinks at least occasionally were lower, at 27.0% and 13.0%, respectively. Of the non-sugary drinks, over half (63.9%) reported that they consumed bottled water, and approximately one-third (32.1%) consumed artificially-sweetened soda at least occasionally. Regular (daily/weekly) consumption was highest for fruit juices (any type) (38.8%), followed by bottled water (37.4%) and soda (28.9%).

Results of bivariate analyses examining associations between consumption of each drink type and demographic characteristics are displayed in Table 1 (gender, age, disadvantage) and Supplementary Table S1 (education, employment, remoteness, parental status, main grocery buyer, and speak English at home). Statistically significant associations were observed between overall SSB consumption and all demographic characteristics. Furthermore, significant associations were observed between gender, age, and education and consumption across all drink types. These relationships are described in more detail in the results of the multivariate analyses where the unique effect of each demographic variable was tested after adjusting for the effect of other demographic variables.

Table 2 displays the statistically significant predictors of past-week SSB consumption. Of the demographic variables, any consumption (i.e., one or more SSBs in the last week versus none) was more likely among males, younger age groups, the most disadvantaged, and participants with the lowest level of education. Similar associations were observed for gender and education when comparing high to moderate consumers. Of the current health, lifestyle, and environment variables included in the analyses, any consumption (versus none) was less likely for those who had received a diagnosis of T2DM. High consumption (vs. moderate) was somewhat more likely among those who had received a diagnosis of depression. No significant associations were observed between consumption and heart disease diagnosis. Any degree of SSB consumption was more likely for those who consumed takeaway food in the past week and those who had soda and fruit juices (any type) available in the home. High consumption was more likely for those consuming takeaway food three or more times in the past week and those who always/almost always had soda and fruit juice available in the home.

Similar patterns of association were observed for males and females when analyzed separately (see Supplementary Table S2), although there were some notable differences. The relationship between consumption and lower education was more consistent across consumption levels for males than for females. Males, but not females, were less likely to consume SSBs after a diagnosis of T2DM, when regularly consuming alcohol, and when artificially-sweetened soda was often available in the home. Having soda but not fruit juice often available in the home was associated with high SSB consumption for males, whereas the reverse was observed for females. High (versus moderate) consumption was more likely among males who were overweight and less likely among females who were overweight.

Table 3 presents separate multivariable analyses for all six drink types. Similar to overall (past-week) SSB consumption, males and those aged 18 to 30 years were most likely to be daily/weekly consumers of soda, energy drinks, sports drinks, and fruit juice. Younger participants were also more likely to be daily/weekly consumers of bottled water. Socio-economic disadvantage was related to soda consumption only. Lower education was associated with consumption of soda, energy drinks, and bottled water. Of the six drink types, only soda consumption varied by remoteness or household grocery buyer responsibilities. Only bottled water consumption varied by employment status. Not speaking English at home was associated with an increased likelihood of consuming energy drinks, fruit juice and bottled water and a decreased likelihood of consuming artificially-sweetened soda. Controlling for demographic differences, daily/weekly soda consumption was associated with an increased likelihood of daily/weekly consumption of all five of the other drink types. Sports drink consumption was also associated with energy drink and fruit juice consumption. Bottled water consumption was associated with fruit juice and artificially-sweetened soda consumption.

With regard to current health measures (Table 3), after controlling for demographic differences, those who were classified as obese were more likely to consume both soda and bottled water. Conversely, those who had ever received a diagnosis of T2DM were less likely to consume soda but were more likely to consume bottled water, artificially-sweetened soda, and energy drinks. Soda consumption was more likely among those who had received a diagnosis of heart disease or depression and those who reported inadequate sleep.

For the behavioral and environmental factors, after controlling for demographic differences, frequent takeaway food consumption was associated with daily/weekly consumption of all six drink types, with strongest associations for soda, sports drinks, fruit juice, and bottled water. Daily/weekly consumption of all alcohol was associated with energy drink consumption, whereas daily/weekly consumption of spirits was associated with bottled water consumption, and to a lesser extent soda. Soda consumption was more likely when it was available in the home. Similar associations between beverage availability in the home and increased consumption were observed for both fruit juice and artificially-sweetened soda. Having artificially-sweetened soda available in the home was associated with a reduced likelihood of consuming soda and fruit juice.

The patterns of association between consumption of each drink type and health and lifestyle variables were similar for males and females when analyzed separately (see Supplementary Tables S3 and S4). There were exceptions where the association was significant for males but not females, or vice versa, but the direction of the relationship was mostly the same. There were some notable differences in results across drink types between males and females. Females, but not males, were more likely to consume multiple rather than single drink types (i.e., soda and bottled water, fruit juices (any type) and bottled water, sports drinks, and artificially-sweetened soda). Receiving a diagnosis of T2DM was associated with decreased soda consumption and increased artificially-sweetened soda consumption for males only and increased energy drink, sports drink, and bottled water consumption for females only. Finally, males who were overweight or obese were more likely to be consumers of bottled water, whereas there was no effect for females.

## 4. Discussion

This study presents a detailed examination of consumption of a selection of pre-packaged non-alcoholic water-based beverages that are widely available for purchase in Australia. The results show that almost half of Australian adults had consumed a drink that was high in free sugar in the past week, with 13.6% having consumed an average of at least one per day. Of all the drink types measured, regular (daily/weekly) consumption of any type of fruit juice was the most prevalent overall, followed closely by bottled water; both at just over one-third of participants. Almost one-third regularly consumed soda, but regular consumption of sports and energy drinks was less common. Nearly one-fifth of participants were regular consumers of artificially-sweetened soda. Regular consumption of each drink type was substantially higher for young adults than for older age groups. It is difficult to compare prevalence of beverage consumption across studies due to differences in methodology and reporting of results. Nevertheless, prevalence of drinks reported in this study were comparable to Australian Health Survey data [33], but were below prevalence reported in the US population [41,55] and for daily SSB consumption in the UK [43] and Norway [39]. A study comparing SSB and fruit juice consumption across 187 countries showed that there is significant heterogeneity both across and within regions, although there are trends of high SSB consumption in the Americas and high fruit juice consumption in the Australasian region [56]. Nevertheless, patterns in consumption by age and sex followed a similar trend across all parts of the world, with consumption highest among males compared to females and decreasing by age [56].

There was significant correlation between consumption of specific drink types such that those who regularly drank soda were also more likely to consume all other drinks types assessed. Furthermore, those who drank any type of fruit juice were more likely to be regular consumers of sports drinks and bottled water. Previous studies have not explored patterns of beverage consumption in this way. The pattern of association between consuming fruit juices (any type), sports drinks, and bottled water observed in this study implies a potential clustering of consumption of beverages that may be viewed as ‘healthier.’ Previous studies have indicated that some drinks are perceived as offering functional and health benefits, notably juice and sports drinks [57,58,59]. Furthermore, any reference to fruit (e.g., fruit flavored soda) can result in elevated perceptions of the healthiness and benefits of the beverage [57], and fruit features are very prevalent on packaging for sugar-containing beverages [58]. Consistent with this, the marketing of sports drinks typically presents the beverage as offering functional or health benefits and/or fulfilling electrolyte or energy deficits [60]. This can lead to a perception by the general public that such beverages are required for recreational physical activity when water usually offers sufficient hydration [61].

In line with previous data from Australia, the US, the UK, and Norway [33,34,35,36,37,38,39,40,41,43], sugary drink consumption was more prevalent among males, young adults, those living in socio-economically disadvantaged areas, and those with lower levels of education. Our study enabled examination by multiple beverage types and these well-established patterns were more pronounced for the ultra-processed drink types. Multivariable analyses demonstrated males had double the odds of being regular soda consumers compared to females, were three times as likely to consume energy drinks, and were five times as likely to consume sports drinks. Patterns of consumption were most striking by age, whereby young adults (18–30 years) were four times more likely to regularly consume soda, eight times more likely to consume sports drinks, and almost 20 times more likely to consume energy drinks regularly. Soda, sports drinks, and energy drinks are routinely marketed depicting youth, masculinity, and sporting prowess [62]. Sports drink and energy drinks, in particular, align their brands with elite sports and extreme sports and sports drinks are ubiquitously promoted via sports sponsorship [62,63,64].

Excess consumption of drinks high in free sugar is problematic because of the known metabolic impacts and health outcomes. Markedly high consumption of soda, sports drinks, and energy drinks in young men is especially important as people gain weight in their teens and early adulthood. Population monitoring shows that young Australian adults have had the largest increase in weight gain between surveys of any population group [30], and strategies that would benefit this demographic group are therefore warranted.

There was no relationship between overall consumption of drinks high in free sugar and either physical activity or BMI. However, consumption of sports drinks was associated with the degree of daily physical activity, likely reflecting the way these drinks are marketed [60]. Those in the obese BMI range were more likely to be regular consumers of soda, although those in either the overweight or obese BMI range were also more likely to drink artificially-sweetened soda and bottled water. Participants who had received a diagnosis of T2DM were less likely to drink SSBs overall and soda in particular, but were more likely to drink bottled waters and somewhat more likely to drink artificially-sweetened beverages. These trends indicate that some people may be consciously trying to make healthier beverage choices. However, emerging evidence indicates that artificially-sweetened beverages may have counter-productive impacts for metabolic health and healthy weight [2,65,66], making consumption of these beverages a poor choice relative to water.

While differences in consumption according to other diagnoses were less pronounced, soda consumption was more prevalent among those who had received a diagnosis of heart disease and depression. Scientific evidence about the cardiovascular impact of high SSB consumption is considerable and increasing [14,15]; however, the relationship is not widely understood by the public [50], creating an opportunity for new public health education campaigns to inform consumers. The relationship between depression and SSB consumption has been reported previously [67], and is supported by a recent meta-analysis reporting significant associations in both cross-sectional and cohort studies [68]. This study contributes to that evidence base, although more in-depth research is needed to understand the reasons for this association and the implications.

Consumption of drinks high in free sugar was strongly predicted by environmental availability and takeaway food consumption. Regular takeaway food consumption was a consistent and strong predictor of overall SSB consumption in general, and also predicted each of the individual beverage types. These drinks are all readily available for sale with takeaway food purchases, and in the instance of quick service restaurants, are often packaged with meals to offer price incentives. Uncoupling artificially-sweetened/regular soda from meal deals or making water the default beverage has gained popularity as a strategy with children’s meals in quick service restaurants [69]. This offers potential as a ‘nudge’ strategy for adults in these environments as well. Availability of soda, juice, and artificially-sweetened soda in the home environment was the strongest predictor of regular consumption for each of these beverages, respectively. There is an important opportunity for health professionals to encourage patients to reduce their sugary consumption by limiting purchases for the home, as well as a need for more direct public education through multi-media campaigns. Major price promotions for sodas and artificially-sweetened sodas are ubiquitous in Australian supermarkets, encouraging purchases and leading to increased availability in the home [70]. The relationships observed here with takeaway food consumption coupled with the known price incentives promoting purchase [71] further the case for interventions to increase the price of SSBs via taxation.

The present study examined data from a large sample of Australian adults. The cross-sectional data means that it is difficult to infer causality from the associations observed. Further limitations were the reliance on self-reporting and the use of brief measures of recall limited to pre-determined drinks, which are not as comprehensive as food diary methodology. The measurement tool used to ascertain beverage consumption was derived from existing measures that have been used in Australian population surveys [47,48,50]. Furthermore, other similar brief self-report beverage intake questionnaires have been shown to be valid and reliable measures of consumption [49,72]. Nevertheless, it is possible that these issues may have produced underestimates of consumption compared to other methods of recording consumption. We also acknowledge that this study was not a comprehensive examination of all drink types that are consumed in Australia. The aim of the study was to assess manufactured water-based drinks, as these products are most relevant to public health interventions designed to reduce free-sugar consumption at the population level [1]. A more comprehensive examination of drink types is an opportunity for future research. The use of telephone survey methodology where only brief verbal descriptions could be provided necessitated the use of simple definitions of drinks (e.g., juice of any type rather than differentiating fruit drinks and 100% fruit juice). Moreover, results from a qualitative study of Australian adults showed that they did not differentiate between 100% juice products and fruit drinks with added sugar; therefore, it was expected that assessing these drinks separately via telephone interview would cause confusion [57].The use of self-reported height and weight to provide a BMI estimate is not as accurate as taking biometric measurements of height and weight, but it is a commonly used approach in studies where resources do not extend to this level of individual assessment, and has been found to approximate BMI in Australian samples [73,74]. Measures used in this study were based on measures used in previous studies (e.g., weight perception [53]), and demonstrate good face validity; however, further validation would be beneficial and would strengthen the results of this study.

In conclusion, many Australian adults regularly consume drinks high in free sugar, with higher consumption rates observed among young adults and males, compared to their demographic counterparts. This pattern persists across all beverage types, including juice. This high consumption foreshadows continued patterns of population weight gain and high burdens of chronic disease if left unchecked. As an overweight nation, reducing SSB and fruit juice consumption is a way to reduce excess free sugars in the diet, and therefore, represents an important policy objective. The popularity of bottled water in Australia, while concerning in other dimensions such as the lack of fluoridation and environmental impact, is promising for positioning tap water as a culturally acceptable alternative to sugar-laden drinks or artificially-sweetened beverages. The popularity of fruit juices warrants serious attention; their very high levels of free sugar does not make them the ‘healthy’ choice that Australia and New Zealand’s front of pack interpretive labelling scheme (health star ratings) has implied.

It is time for reconsideration of dietary guidelines and labelling reform in Australia, which is beginning to emerge internationally. The Australian Government is currently considering introducing added sugar labelling. Adopting added sugar labelling, and improving and making the interpretive labelling mandatory, would improve consumer information and guidance. Australia has produced effective public education campaigns in a few states, with demonstrated capacity to reduce SSB consumption and increase water consumption, thus, a nation-wide scale-up of such public education campaigns would yield tangible returns. It is also time for Australia to put a heath levy on SSBs to encourage the consumption reductions demonstrated by SSB taxes internationally and to generate income to fund complementary initiatives such as public education.

## Figures and Tables

**Table 1:** Overall consumption for sugary drinks and individual drink types, and associations between consumption of each drink type with gender, age, and socio-economic disadvantage (*n* = 3430).

	Overall		Gender		Age (years)				Disadvantage (Deciles)		
			Male	Female	18–30	31–45	46–60	61+	Most (1–3)	Mid (4–7)	Least (8–10)
Drink Type	%	95% CI	%	%	%	%	%	%	%	%	%
**Sugar-sweetened beverage consumption (past week)**			*p* < 0.001		*p* < 0.001				*p* = 0.001		
High (7 or more times)	13.6	12.5–14.7	**19.3**	**8.2**	**17.2**	**16.3**	12.5	**8.9**	**17.6**	13.7	**11.5**
Moderate (1 to 6 times)	33.7	32.1–35.3	**36.6**	**31.4**	**49.9**	**38.4**	**28.6**	**20.5**	34.2	35.3	32.5
None	52.1	50.4–53.8	**44.2**	**60.4**	**32.9**	**45.3**	**59.0**	**70.6**	**48.1**	51.0	**55.9**
**Soda**			*p* < 0.001		*p* < 0.001				*p* = 0.003		
Daily or weekly	28.9	27.4–30.5	**38.4**	**19.9**	**46.7**	**33.1**	**23.5**	**14.8**	**34.1**	29.1	**26.4**
Monthly or less	34.7	33.1–36.3	**32.7**	**36.7**	**38.3**	**38.5**	36.1	**26.4**	**29.8**	35.8	36.0
Never	36.2	34.6–37.8	**28.9**	**43.4**	**15.0**	**28.4**	**40.4**	**58.8**	36.0	35.2	37.5
**Energy drink**			*p* < 0.001		*p* < 0.001				*p* = 0.200		
Daily or weekly	4.2	3.5–4.9	**6.9**	**1.7**	**11.3**	4.2	**1.6**	**0.6**	3.3	4.9	4.0
Monthly or less	8.8	7.9–9.8	**12.3**	**5.5**	**17.9**	**11.4**	**4.9**	**1.9**	9.5	9.5	7.8
Never	86.9	85.8–88.0	**80.9**	**92.8**	**70.8**	**84.5**	**93.5**	**97.5**	87.2	85.6	88.1
**Sports drink**			*p* < 0.001		*p* < 0.001				*p* = 0.060		
Daily or weekly	8.1	7.2–9.0	**14.1**	**2.3**	**19.2**	7.3	**5.0**	**2.0**	7.4	8.9	7.6
Monthly or less	18.9	17.6–20.2	**25.5**	**12.5**	**31.2**	**24.8**	**15.1**	**5.8**	16.9	17.9	21.2
Never	72.8	71.3–74.3	**60.4**	**85.2**	**49.6**	**67.8**	**79.9**	**92.2**	75.6	73.2	71.2
**Fruit juices (any type)**			*p* < 0.001		*p* < 0.001				*p* = 0.379		
Daily or weekly	38.8	37.2–40.4	**46.9**	**31.0**	**49.0**	38.3	**33.7**	**35.1**	40.2	38.7	38.5
Monthly or less	29.0	27.5–30.5	**26.1**	**31.9**	27.6	**32.5**	29.8	**26.1**	26.4	28.7	30.6
Never	32.1	30.5–33.7	**27.0**	**37.1**	**23.3**	**29.2**	**36.5**	**38.8**	33.3	32.6	30.9
**Artificially-sweetened soda**			*p* = 0.149		*p* < 0.001				*p* = 0.242		
Daily or weekly	18.1	16.8–19.4	19.1	17.0	18.7	20.3	20.0	**14.1**	18.8	18.6	17.4
Monthly or less	14.0	12.9–15.2	14.5	13.5	**19.5**	**16.5**	**11.3**	**9.4**	13.0	12.9	15.7
Never	67.9	66.3–69.4	66.4	69.4	**61.8**	**63.2**	68.7	**76.5**	68.2	68.5	66.9
**Pre-packaged bottled water**			*p* < 0.001		*p* < 0.001				*p* = 0.001		
Daily or weekly	37.4	35.8–39.0	**39.8**	**35.3**	**48.6**	40.3	36.9	**25.2**	38.6	37.2	37.4
Monthly or less	26.2	24.7–27.7	**22.9**	**29.6**	24.8	**29.5**	27.9	**22.6**	**20.5**	26.8	**28.7**
Never	36.1	34.5–37.7	37.3	35.1	**26.5**	**30.1**	35.3	**52.3**	**40.9**	36.0	**33.9**

Notes: *p*-values are the result of chi-square tests. Bold cells are statistically significant at *p* < 0.05 (Based on adjusted standardized residuals). ‘Don’t know’ responses are excluded, thus overall percentages do not always add up to 100%. Mid indicates moderate level of disadvantage. CI = Confidence Interval.

**Table 2:** Hierarchical logistic regression analyses examining demographic, health, behavior, and environmental predictors of past week sugar-sweetened beverages consumption.

	Sugar-Sweetened Beverage Consumption			
	Any vs. None		High vs. Moderate	
	OR	95% CI	OR	95% CI
**Block 1**				
Gender (ref = female)				
Male	1.88 ***	1.59–2.22	1.86 ***	1.44–2.40
Age (ref = 61 years and over)				
18–30 years	4.54 ***	3.53–5.82	0.82	0.56–1.18
31–45 years	3.13 ***	2.37–4.14	1.13	0.74–1.72
46–60 years	1.71 ***	1.32–2.20	1.08	0.73–1.62
Disadvantage decile (ref = least (8–10))				
Most (1–3)	1.65 ***	1.31–2.08	1.21	0.88–1.68
Mid (4–7)	1.23 *	1.02–1.48	0.94	0.72–1.24
Education (ref = bachelor degree or higher)				
Secondary school or less	1.38 **	1.12–1.71	2.21 ***	1.62–3.02
Some tertiary or finished vocational	1.21 *	1.00–1.47	1.78 ***	1.33–2.37
**Block 2 (Controlling for block 1)**				
**Current health**				
BMI perception (ref = Acceptable weight or underweight)				
Overweight	1.28 *	1.02–1.61	0.76	0.55–1.06
Ever had any of the following conditions (ref = No)				
Type 2 Diabetes	0.56 **	0.36–0.85	1.27	0.68–2.38
Depression	1.15	0.90–1.47	1.58 **	1.17–2.14
**Behavior**				
Takeaway food past 7 days (ref = not at all)				
About 1–2 times	2.13 ***	1.76–2.58	1.25	0.92–1.71
About 3 or more times	6.68 ***	4.54–9.84	2.50 ***	1.68–3.72
Alcohol consumption (ref = no)				
Consume alcohol daily or weekly	0.81 *	0.67–0.99	0.81	0.62–1.06
Consume spirits daily or weekly	1.39 *	1.01–1.92	1.54 *	1.05–2.27
**Environment**				
Soda available in the home (ref = never)				
Always or almost always	6.23 ***	4.65–8.35	1.81 **	1.21–2.71
Sometimes or seldom	2.62 ***	2.09–3.28	0.94	0.65–1.38
Artificially-sweetened soda available in the home (ref = never)				
Always or almost always	0.70 **	0.53–0.91	1.08	0.77–1.51
Sometimes or seldom	1.06	0.87–1.30	0.82	0.62–1.08
Fruit juice available in the home (ref = never)				
Always or almost always	3.96 ***	2.96–5.31	1.98 **	1.23–3.19
Sometimes or seldom	1.61 **	1.22–2.13	1.20	0.74–1.93

Note: Analyses controlled for ‘typical consumption.’ Ref indicates reference category. Variables included in the model but not statistically significant at *p* < 0.05: Employment; Remoteness; Parental status; Grocery buyer; Speak English at home; Body Mass Index (BMI); Ever been diagnosed with: Heart disease, Arthritis or gout, or lung condition such as asthma or Chronic Obstructive Pulmonary Disease (COPD); Physical activity; Sleep; Shift work. High consumption refers to 7 or more sugary drinks consumed in the past week, and moderate consumption refers to 1–6 sugary drinks consumed in the past week. Any consumption refers to consumption of one or more sugar-sweetened beverages in the past week. * *p* < 0.05; ** *p* < 0.01; *** *p* < 0.001. OR = Odds Ratio; CI = Confidence Interval.

**Table 3:** Hierarchical logistic regression analyses examining demographic characteristics (block 1), other drink types (block 2), and current health, behavior, and environment variables (block 3) as predictors of individual types of beverage consumption (Daily/weekly versus monthly or less).

	Soda		Energy		Sports		Fruit Juices (Any Type)		Artificially-Sweetened Soda		Pre-Packaged Bottled Water	
	OR	95% CI	OR	95% CI	OR	95% CI	OR	95% CI	OR	95% CI	OR	95% CI
**Block 1**												
Gender (ref = female)												
Male	2.08 ***	1.75–2.47	3.03 ***	1.93–4.76	5.19 ***	3.63–7.41	1.83 ***	1.56–2.14	1.17	0.97-1.42	1.05	0.90–1.22
Age (ref = 61 years and over)												
18–30 years	4.70 ***	3.62–6.10	19.77 ***	6.94–56.34	8.25 ***	4.83–14.10	1.56 ***	1.25–1.94	1.33	1.00–1.78	2.26 ***	1.80–2.84
31–45 years	3.37 ***	2.50–4.56	7.21 **	2.34–22.22	3.04 ***	1.65–5.61	1.03	0.80–1.34	1.58 **	1.14–2.19	1.60 ***	1.23–2.08
46–60 years	2.05 ***	1.55–2.72	2.83	0.87–9.16	2.30 **	1.25–4.24	0.99	0.78–1.25	1.48 **	1.10–1.99	1.42 **	1.12–1.80
Disadvantage (ref = least disadvantage (deciles 8–10))												
Most disadvantage (Deciles 1–3)	1.65 ***	1.30–2.08	0.57	0.32–1.02	1.08	0.72–1.61	1.21	0.98–1.50	1.28	0.98–1.67	1.13	0.92–1.41
Mid disadvantage (Decile 4–7)	1.11	0.91–1.34	0.87	0.57–1.33	1.19	0.87–1.62	1.04	0.88–1.24	1.22	0.99–1.52	0.99	0.83–1.17
Education (ref = completed bachelor degree or higher)												
Secondary school or less	1.63 ***	1.30–2.08	3.63 ***	2.12–6.22	0.99	0.68–1.44	0.92	0.76–1.12	0.91	0.71–1.16	1.33 **	1.09–1.62
Some tertiary or finished vocational	1.46 ***	1.20–1.78	2.98 ***	1.80–4.94	1.25	0.90–1.74	0.95	0.80–1.14	0.77*	0.62–0.97	1.36 **	1.14–1.63
Employment (ref = currently working)												
Not currently working	1.07	0.89–1.30	0.56 *	0.36–0.90	0.72	0.52–1.01	1.18	1.00–1.41	0.83	0.66–1.03	0.67 ***	0.56–0.80
Remoteness (ref = major cities)												
Inner regional	0.69 **	0.55–0.87	1.23	0.73–2.06	0.77	0.52–1.15	0.85	0.70–1.04	0.89	0.70–1.14	0.85	0.70–1.04
Outer regional, remote, very remote	1.07	0.81–1.41	1.59	0.87–2.90	1.24	0.79–1.94	0.83	0.64–1.08	0.75	0.54–1.04	0.94	0.73–1.22
Grocery buyer (ref = yes or shared responsibility)												
No	1.37 **	1.14–1.65	0.62 *	0.41–0.93	1.21	0.91–1.61	1.10	0.92–1.31	1.05	0.84–1.30	0.97	0.81–1.16
Speak English at home (ref = yes)												
No	0.98	0.74–1.32	2.50 **	1.49–4.18	1.16	0.76–1.78	2.07 ***	1.57–2.71	0.51 **	0.34–0.76	1.75***	1.34–2.29
**Block 2 (Controlling for block 1)**												
Daily or weekly consumption of... (ref = no)												
Soda			3.06 ***	1.98–4.71	2.32 ***	1.72–3.12	2.34 ***	1.97–2.78	1.88 ***	1.52–2.31	1.27**	1.07–1.51
Energy drinks	2.99 ***	1.96–4.57			3.24 ***	2.11–4.99	1.37	0.93–2.03	0.66	0.40–1.09	1.02	0.70–1.49
Sports drinks	2.28 ***	1.70–3.06	3.10 ***	2.01–4.77			1.45 *	1.09–1.93	1.30	0.94–1.79	1.01	0.76–1.33
Fruit juices (any type)	2.36 ***	1.99–2.80	1.42	0.95–2.13	1.51 **	1.13–2.01			1.03	0.84–1.24	1.27**	1.08–1.48
Artificially-sweetened soda	1.91 ***	1.55–2.35	0.66	0.39–1.12	1.29	0.92–1.80	1.03	0.85–1.25			1.34**	1.11–1.62
Pre-packaged bottled water	1.27 **	1.07–1.52	1.03	0.70–1.52	1.00	0.76–1.33	1.26 **	1.08–1.48	1.34 **	1.11–1.62		
**Block 3 (Controlling for blocks 1 & 2)**												
**Current health**												
BMI (ref = Underweight/healthy-up to 25)												
Overweight (25.1–29.9)	0.97	0.75–1.25	1.55	0.94–2.57	1.44	1.00–2.06	0.89	0.70–1.12	1.36 *	1.01–1.84	1.32**	1.08–1.61
Obese (30–75)	1.61 **	1.16–2.23	1.58	0.78–3.23	1.18	0.71–1.98	0.96	0.71–1.31	1.61 *	1.11–2.34	1.45 **	1.12–1.89
Don’t know	1.9 3*	1.13–3.29	0.38	0.06–2.29	1.07	0.40–2.83	0.70	0.41–1.17	1.38	0.75–2.53	1.10	0.71–0.70
Ever had any of the following conditions (ref = No)												
Type 2 Diabetes	0.46 **	0.28–0.76	4.23*	1.40–12.75	1.21	0.52–2.86	1.06	0.71–1.58	1.66 *	1.04–2.63	1.63 **	1.17–2.27
Heart Disease	1.60 *	1.04–2.48	0.53	0.09–3.03	0.62	0.25–1.56	0.98	0.66–1.45	1.07	0.65–1.76	0.69 *	0.48–1.00
Depression	1.30 *	1.02–1.67	1.22	0.73–2.03	1.15	0.79–1.67	0.87	0.69–1.11	0.89	0.67–1.19	0.93	0.76–1.14
**Behavior**												
Takeaway food past 7 days (ref = not at all)												
About 1–2 times	2.12 ***	1.71–2.63	1.90 *	1.03–3.50	1.27	0.87–1.85	1.31 **	1.07–1.60	1.20	0.93–1.55	1.10	0.93–1.30
About 3 or more times	3.88 ***	2.76–5.43	2.38 *	1.17–4.86	2.70 ***	1.71–4.26	1.62 **	1.15–2.27	1.65 *	1.10–2.48	1.52 **	1.14–2.02
Alcohol consumption (ref = no)												
Consume alcohol daily or weekly	0.86	0.70–1.06	2.72 ***	1.70–4.37	1.18	0.86–1.63	1.12	0.92–1.36	0.99	0.77–1.26	1.14	0.97–1.34
Consume spirits daily or weekly	1.43 *	1.04–1.96	1.66	1.00–2.77	1.44	0.95–2.18	1.13	0.83–1.54	1.52 *	1.05–2.19	1.55 **	1.20–2.01
Physical activity past 7 days (ref = none)												
Some days (1 to 6 days)	0.88	0.62–1.27	1.04	0.41–2.63	2.05	0.91–4.63	1.13	0.80–1.61	0.95	0.63–1.45	0.75	0.56–1.01
Every day (7 days)	0.84	0.58–1.22	0.80	0.31–2.09	2.89 *	1.27–6.60	1.06	0.74–1.53	0.69	0.44–1.07	0.85	0.63–1.15
Sleep (ref = 6 h or less)												
7 to 8 h	0.76 *	0.62–0.94	0.66	0.42–1.02	1.10	0.80–1.52	0.91	0.75–1.11	1.00	0.78–1.28	1.03	0.87–1.22
9 or more hours	0.87	0.58–1.22	1.30	0.63–2.69	0.95	0.52–1.75	1.15	0.80–1.66	1.00	0.62–1.60	1.02	0.74–1.40
**Environment**												
Soda available in the home (ref = never)												
Always or almost always	13.07 ***	9.36–18.24	1.31	0.62–2.80	0.93	0.56–1.55	0.70 *	0.52–0.95	0.21 ***	0.14–0.30	1.13	0.88–1.47
Sometimes or seldom	3.99 ***	3.00–5.31	1.56	0.81–3.04	1.30	0.85–1.99	0.97	0.77–1.21	0.43 ***	0.32–0.57	1.14	0.94–1.38
Artificially-sweetened soda available in the home (ref = never)												
Always or almost always	0.47 ***	0.35–0.65	1.13	0.59–2.15	1.14	0.73–1.79	0.67 **	0.50–0.90	71.41 ***	48.56–105.02	1.09	0.85–1.41
Sometimes or seldom	1.03	0.83–1.28	0.94	0.59–1.50	0.94	0.67–1.32	0.88	0.71–1.08	11.76 ***	8.30–16.65	1.23 *	1.03–1.46
Fruit juice available in the home (ref = never)												
Always or almost always	1.68 **	1.17–2.42	1.10	0.46–2.63	1.27	0.71–2.26	81.41 ***	49.46–134.01	0.53 **	0.36–0.79	1.29	0.98–1.70
Sometimes or seldom	1.20	0.86–1.66	0.95	0.43–2.11	0.94	0.56–1.59	10.13 ***	6.23–16.46	0.59 **	0.42–0.83	1.15	0.92–1.45

Note: ref indicates reference category. Variables included in each model but not statistically significant at *p* < 0.05 for any drink type: Parental status; Body Mass Index (BMI) perception; Shiftwork and Ever been diagnosed with arthritis or gout or lung condition such as asthma or Chronic Obstructive Pulmonary Disease (COPD). * *p* < 0.05; ** *p* < 0.01; *** *p* < 0.001. OR = Odds Ratio; CI = Confidence Interval.

## Supplementary Materials

The following are available online at https://www.mdpi.com/2072-6643/12/3/817/s1.
