# Supplementary material for: Consumption of Sugar-Sweetened Beverages, Juice, Artificially-Sweetened Soda and Bottled Water: An Australian Population Study
Source: Nutrients. 2020 Mar 19;12(3):817. doi: 10.3390/nu12030817 (PMC7146120; doi:10.3390/nu12030817)
Supplement: Supplementary file 1 [file nutrients-12-00817-s001.pdf]

Supplementary tables

Table S1. Associations between demographics and consumption of sugar–sweetened beverages and individual drink types.

		Education			Employment		Area			Parent of child under 18		Main grocery buyer		Speak English at home	
	Overall	High school or less	Vocation or partial university	Bachelor degree or higher	Working	Not currently working	Major cities	Inner regional	Outer regional or beyond	Yes	No	Yes–all or shared	No	Yes	No
Drink type	%	%	%	%	%	%	%	%	%	%	%	%	%	%	%
**SSB (past week)**			$p < 0.001$		$p < 0.001$			$p = 0.031$		$p = 0.048$		$p < 0.001$		$p = 0.024$	
7 or more times	13.6	**17.1**	**16.2**	**8.9**	**14.6**	**12.1**	12.9	13.6	**18.8**	**15.5**	**12.8**	**11.8**	**18.5**	13.7	13.1
1 to 6 times	33.7	32.4	34.2	35.3	**36.4**	**30.4**	34.9	31.9	31.5	34.8	33.6	**32.0**	**39.2**	**33.2**	**41.2**
none	52.1	50.5	**49.6**	**55.8**	**49.0**	**57.5**	52.1	54.5	49.7	**49.7**	**53.6**	**56.2**	**42.4**	**53.1**	**45.6**
**Soda**			$p < 0.001$		$p < 0.001$			$p = 0.005$		$p < 0.001$		$p < 0.001$		$p = 0.001$	
Daily or weekly	28.9	**32.2**	**32.5**	**23.8**	**31.3**	**25.6**	29.9	**24.2**	32.5	30.0	28.5	**24.4**	**40.9**	**28.3**	**36.6**
Monthly or less	34.7	**32.1**	33.6	**37.9**	**36.9**	**31.5**	35.1	33.9	33.0	**40.0**	**32.5**	35.2	33.8	34.5	37.0
Never	36.2	35.7	33.9	**38.3**	**31.8**	**42.9**	**35.0**	**41.9**	34.5	**30.1**	**38.9**	**40.3**	**25.3**	**37.2**	**26.4**
**Energy drink**			$p < 0.001$		$p < 0.001$			$p = 0.427$		$p = 0.599$		$p < 0.001$		$p < 0.001$	
Daily or weekly	4.2	**5.5**	**5.5**	**2.2**	**5.4**	**2.4**	4.3	3.7	4.9	3.7	4.4	**3.6**	**5.8**	**3.8**	**9.8**
Monthly or less	8.8	8.2	8.6	9.6	**10.4**	**6.4**	9.2	7.2	9.5	9.2	8.7	**8.1**	**11.0**	**8.5**	**13.0**
Never	86.9	86.3	85.9	88.2	**84.1**	**91.2**	86.5	89.1	85.6	87.1	86.9	**88.3**	**83.3**	**87.8**	**77.2**

**Sports drink**			$p = 0.016$		$p < 0.001$			$p = 0.014$		$p = 0.329$		$p < 0.001$		$p = 0.007$	
Daily or weekly	8.1	7.6	**10.1**	**7.0**	**10.2**	**5.0**	8.6	**5.8**	9.5	7.5	8.4	**6.1**	**13.8**	**7.8**	**12.7**
Monthly or less	18.9	17.3	18.3	**20.7**	**22.4**	**13.6**	**19.9**	16.7	16.4	20.3	18.3	**16.5**	**25.5**	18.8	20.7
Never	72.8	75.1	71.6	72.4	**67.4**	**81.4**	**71.5**	**77.5**	74.1	72.2	73.2	**77.5**	**60.8**	**73.5**	**66.5**
**Fruit juices (any type)**			$p = 0.001$		$p = 0.111$			$p = 0.041$		$p = 0.043$		$p < 0.001$		$p < 0.001$	
Daily or weekly	38.8	37.9	39.2	39.5	38.7	39.0	**40.5**	**35.0**	35.8	39.0	38.8	**36.0**	**46.2**	**37.0**	**60.4**
Monthly or less	29.0	**25.2**	29.2	**31.5**	30.2	27.2	28.8	29.1	29.8	**31.5**	**27.9**	29.4	28.2	**29.5**	**23.6**
Never	32.1	**36.9**	31.6	**29.0**	31.1	33.8	**30.7**	**35.9**	34.4	**29.5**	**33.3**	**34.6**	**25.6**	**33.5**	**16.0**
**Artificially–sweetened soda**			$p < 0.001$		$p < 0.001$			$p = 0.131$		$p = 0.911$		$p = 0.444$		$p < 0.001$	
Daily or weekly	18.1	18.1	16.6	19.6	**19.9**	**15.4**	18.6	17.9	15.5	18.5	17.9	17.9	18.4	**18.5**	**13.8**
Monthly or less	14.0	**11.2**	13.3	**16.7**	**15.7**	**11.5**	14.8	12.7	11.5	13.9	14.1	13.6	15.1	**13.3**	**21.7**
Never	67.9	**70.7**	**70.0**	**63.7**	**64.4**	**73.1**	66.7	69.4	73.0	67.6	68.0	68.5	66.5	68.2	64.5
**Pre–packaged bottled water**			$p < 0.001$		$p < 0.001$			$p = 0.004$		$p < 0.001$		$p = 0.028$		$p < 0.001$	
Daily or weekly	37.4	36.8	**40.2**	35.7	**42.2**	**30.0**	**38.8**	**33.3**	36.7	**40.1**	**36.3**	**36.4**	**40.5**	**36.2**	**52.9**
Monthly or less	26.2	**20.6**	25.6	**31.0**	27.3	24.9	26.9	25.8	22.3	**29.3**	**25.0**	26.1	26.7	**26.8**	**19.7**
Never	36.1	**42.5**	34.3	**33.3**	**30.5**	**45.0**	**34.3**	**40.9**	41.0	**30.6**	**38.7**	**37.5**	**32.8**	**37.0**	**27.4**

Note: *p*–values are the result of chi–square tests. Bold cells are statistically significant (Based on adjusted standardised residuals). 'Don't know' responses excluded so overall percentages do not always add up to 100%. Mid indicates moderate level of disadvantage.

Table S2: Hierarchical logistic regression analyses examining demographic, health, behavior and environmental predictors of past week sugar–sweetened beverages consumption for males and females separately.

	Sugar–sweetened beverage consumption							
	Any vs None				High vs Moderate			
	Males (n = 1689)		Females (n = 1741)		Males (n = 935)		Females (n = 687)	
Prevalence	%		%		%		%	
None	43.9		60.1					
1 or more times	55.4		39.5					
1–6 times					65.5		79.3	
7+ times					34.5		20.7	
Logistic regression	**OR**	**95% CI**	**OR**	**95% CI**	**OR**	**95% CI**	**OR**	**95% CI**
Block 1								
**Age (ref= 61 years and over)**								
18–30 years	5.06***	3.63–7.07	3.89***	2.60–5.81	0.95	0.60–1.51	0.56	0.28–1.13
31–45 years	2.87***	1.97–4.20	3.54***	2.32–5.43	1.13	0.67–1.91	1.24	0.60–2.57
46–60 years	1.83**	1.28–2.63	1.60*	1.11–2.31	1.08	0.64–1.83	1.16	0.62–2.19
**Disadvantage decile (ref=least (8–10))**								
Most (1–3)	1.53*	1.11–2.11	1.81***	1.30–2.53	1.21	0.80–1.83	1.17	0.67–2.03
Mid (4–7)	1.07	0.82–1.39	1.41*	1.08–1.84	0.90	0.64–1.28	0.96	0.60–1.54
**Education (ref=bachelor degree or higher)**								
Secondary school or less	1.39*	1.03–1.88	1.40*	1.03–1.90	2.71***	1.81–4.03	1.45	0.85–2.48
Some tertiary or finished vocational	1.42*	1.09–1.86	1.05	0.79–1.39	1.94***	1.34–2.81	1.73*	1.06–2.83
Block 2 (Controlling for block 1)								
Current health								
**BMI (ref=Underweight/healthy–up to 25)**								
Overweight (25.1–29.9)	0.87	0.63–1.19	1.00	0.69–1.44	1.83**	1.23–2.72	0.50*	0.26–0.97
Obese (30–75)	1.09	0.71–1.68	1.41	0.90–2.22	1.59	0.93–2.74	0.72	0.34–1.49
Don't know	0.52	0.20–1.36	0.89	0.44–1.80	1.05	0.30–6.63	1.01	0.39–2.62
**Ever had any of the following conditions (ref=No)**								
Type 2 Diabetes	0.39**	0.21–0.72	0.70	0.38–1.27	0.70	0.27–1.77	2.42	0.95–6.20
Behavior								
**Takeaway food past 7 days (ref=not at all)**								
About 1–2 times	2.25***	1.70–2.99	2.13***	1.63–2.79	1.54*	1.01–2.36	1.16	0.70–1.93
About 3 or more times	6.27***	3.88–10.14	10.25***	4.95–21.23	3.10***	1.87–5.15	2.29*	1.10–4.75
**Alcohol consumption (ref=no)**								
Consume alcohol daily or weekly	0.69**	0.52–0.91	0.92	0.69–1.24	0.84	0.60–1.18	0.76	0.46–1.24
Consume spirits daily or weekly	1.18	0.78–1.80	1.78*	1.08–2.93	1.76*	1.10–2.81	0.93	0.42–2.02
Environment								
**Soda available in the home (ref=never)**								
Always or almost always	7.30***	4.83–11.02	5.39***	3.50–8.30	2.12**	1.24–3.62	1.68	0.85–3.29
Sometimes or seldom	3.03***	2.20–4.17	2.26***	1.63–3.14	1.22	0.74–2.01	0.65	0.35–1.21
**Artificially–sweetened soda available in the home (ref=never)**								
Always or almost always	0.61*	0.42–0.89	0.87	0.58–1.31	1.13	0.73–1.74	1.14	0.63–2.06
Sometimes or seldom	0.97	0.72–1.29	1.23	0.91–1.65	0.91	0.64–1.30	0.75	0.45–1.25
**Fruit juice available in the home (ref=never)**								
Always or almost always	3.64***	2.36–5.60	4.97***	3.24–7.63	1.55	0.84–2.84	3.97**	1.61–9.82
Sometimes or seldom	1.61*	1.07–2.44	1.70**	1.15–2.53	0.91	0.49–1.67	2.20	0.89–5.41

Note: Analyses controlled for 'typical consumption'. Ref indicates reference category. Variables included in the model but not statistically significant at $p<0.05$: Employment; Remoteness; Parental status; Grocery buyer; Speak English at home; BMI perception; Ever been diagnosed with: Heart disease, Arthritis or gout, Depression or lung condition such as asthma or COPD; Physical activity; Sleep; Shift work. High consumption refers to 7 or more sugary drinks consumed in the past week, and moderate consumption refers to 1–6 sugary drinks consumed in the past week. Any consumption refers to consumption of one or more sugar–sweetened beverages in the past week. *$p<0.05$; **$p<0.01$; ***$p<0.001$.

Table S3: Hierarchical logistic regression analyses examining demographic characteristics (block 1), other drink types (block 2), and current health, behavior and environment variables (block 3) as predictors of soda, energy drink and sports drink consumption (Daily/weekly versus monthly or less) for males and females separately.

	**Soda**				**Energy drinks**				**Sports drinks**			
	**Male**		**Female**		**Male**		**Female**		**Male**		**Female**	
	**OR**	**95% CI**	**OR**	**95% CI**	**OR**	**95% CI**	**OR**	**95% CI**	**OR**	**95% CI**	**OR**	**95% CI**
**(Prevalence of daily/weekly consumption)**	**(38.3%)**		**(19.9%)**		**(6.8%)**		**(1.7%)**		**(14.4%)**		**(2.3%)**	
Block 1												
**Age (ref= 61 years and over)**												
18–30 years	4.51***	3.24–6.27	4.90***	3.12–7.69	16.73***	5.03–55.61	30.71**	3.54–266.49	8.79***	4.90–15.79	5.83*	1.43–23.79
31–45 years	3.06***	2.08–4.49	4.09***	2.48–6.74	7.49**	2.09–26.91	4.22	0.39–46.14	3.02**	1.54–5.90	3.94	0.84–18.44
46–60 years	2.21***	1.52–3.21	1.90**	1.22–2.97	3.05	0.79–11.83	2.09	0.19–22.79	2.13*	1.07–4.22	2.70	0.65–11.13
**Disadvantage (ref=least disadvantage (deciles 8–10))**												
Most disadvantage (Deciles 1–3)	1.78***	1.31–2.43	1.46*	1.02–2.11	0.54	0.28–1.07	0.66	0.20–2.19	1.07	0.69–1.66	0.94	0.32–2.76
Mid disadvantage (Decile 4–7)	1.13	0.88–1.46	1.10	0.82–1.48	0.92	0.57–1.49	0.77	0.31–1.90	1.03	0.73–1.46	1.99	0.94–4.21
**Education (ref=completed bachelor degree or higher)**												
Secondary school or less	1.71***	1.28–2.29	1.61**	1.14–2.27	2.98***	1.63–5.43	7.87**	2.15–28.83	1.05	0.70–1.59	0.70	0.25–1.93
Some tertiary or finished vocational	1.44**	1.10–1.87	1.58**	1.16–2.16	2.34**	1.32–4.12	6.29**	1.86–21.28	1.24	0.86–1.79	1.65	0.79–3.45
**Employment (ref=currently working)**												
Not currently working	1.13	0.87–1.48	1.01	0.75–1.34	0.52*	0.30–0.91	0.57	0.23–1.37	0.83	0.57–1.20	0.49	0.22–1.09
**Remoteness (ref=major cities)**												
Inner regional	0.71*	0.53–0.96	0.66*	0.46–0.94	1.23	0.68–2.24	1.41	0.49–4.05	0.73	0.47–1.15	1.08	0.46–2.54
Outer regional, remote, very remote	0.90	0.63–1.30	1.37	0.91–2.07	1.54	0.76–3.09	1.93	0.58–6.40	1.33	0.82–2.18	0.98	0.29–3.28
**Grocery buyer (ref=yes or shared responsibility)**												
No	1.29*	1.04–1.61	1.56*	1.08–2.27	0.65	0.42–1.01	0.59	0.20–1.77	1.15	0.85–1.56	1.46	0.64–3.36
**Speak English at home (ref=yes)**												
No	1.10	0.76–1.57	0.83	0.50–1.38	2.94***	1.69–5.11	0.86	0.14–5.18	1.03	0.64–1.65	2.33	0.88–6.14
Block 2 (Controlling for block 1)												
**Daily or weekly consumption of... (ref=no)**												

Soda					2.54***	1.56–4.15	5.60***	2.15–14.59	2.19***	1.58–3.03	3.06**	1.47–6.34
Energy drinks	2.53***	1.57–4.10	5.46***	2.24–13.28					3.17***	1.99–5.03	7.03**	2.11–23.45
Sports drinks	2.20***	1.59–3.04	3.06**	1.49–6.30	3.24***	2.03–5.17	5.31*	1.47–19.24				
Fruit juices (any type)	2.20***	1.76–2.76	2.58***	1.96–3.38	1.49	0.94–2.38	1.07	0.43–2.66	1.58**	1.15–2.17	1.19	0.58–2.44
Artificially–sweetened soda	1.80***	1.37–2.38	2.10***	1.53–2.88	0.74	0.41–1.31	0.51	0.13–2.02	1.06	0.72–1.56	2.75**	1.35–5.59
Pre–packaged bottled water	1.17	0.93–1.47	1.47**	1.12–1.93	1.21	0.78–1.89	0.48	0.20–1.20	0.95	0.69–1.30	1.48	0.76–2.92
Block 3 (Controlling for blocks 1 & 2)												
Current health												
**BMI (ref=Underweight/healthy–up to 25)**												
Overweight (25.1–29.9)	0.88	0.64–1.21	1.10	0.71–1.72	1.45	0.83–2.54	2.92	0.63–13.54	1.47	0.99–2.19	1.20	0.43–3.35
Obese (30–75)	1.45	0.95–2.23	1.75*	1.02–3.00	1.76	0.77–3.99	3.62	0.52–25.04	1.20	0.67–2.13	0.97	0.24–3.85
Don’t know	2.56*	1.10–5.96	1.57	0.75–3.30	–	–	3.29	0.26–41.49	0.91	0.26–3.19	0.78	0.11–5.54
**Ever had any of the following conditions (ref=No)**												
Type 2 Diabetes	0.44*	0.23–0.86	0.48	0.22–1.07	2.53	0.56–11.40	9.96*	1.22–81.47	0.35	0.08–1.50	7.09**	1.91–26.32
Heart Disease	1.60	0.94–2.73	1.31	0.59–2.94	0.65	0.11–3.99	–	–	0.60	0.21–1.68	3.49	0.48–25.39
Depression	1.14	0.80–1.62	1.39	0.97–2.01	1.02	0.54–1.96	0.98	0.32–3.04	1.00	0.64–1.58	1.20	0.50–2.86
Behaviour												
**Takeaway food past 7 days (ref=not at all)**												
About 1–2 times	2.05***	1.52–2.78	2.26***	1.62–3.14	1.92	0.93–3.97	2.60	0.65–10.37	1.47	0.94–2.30	0.64	0.25–1.61
About 3 or more times	3.38***	2.21–5.19	5.01***	2.78–9.03	2.49*	1.09–5.67	13.6*	1.84–100.44	2.82***	1.67–4.75	2.79	0.91–8.52
**Alcohol consumption (ref=no)**												
Consume alcohol daily or weekly	0.85	0.65–1.11	0.84	0.59–1.18	2.44**	1.42–4.20	3.47*	1.04–11.6	1.26	0.88–1.80	0.75	0.30–1.85
Consume spirits daily or weekly	1.45	0.97–2.18	1.38	0.81–2.34	1.54	0.85–2.78	2.14	0.52–8.82	1.47	0.93–2.33	2.19	0.65–7.37
**Physical activity past 7 days (ref=none)**												
Some days (1 to 6 days)	1.04	0.63–1.73	0.74	0.43–1.27	0.59	0.22–1.58	–	–	2.12	0.85–5.29	4.55	0.43–47.69
Every day (7 days)	1.07	0.63–1.80	0.63	0.36–1.10	0.55	0.20–1.53	–	–	2.90*	1.15–7.31	8.91	0.83–95.7
**Sleep (ref=6 hours or less)**												
7 to 8 hours	0.87	0.66–1.14	0.65*	0.46–0.91	0.76	0.46–1.25	0.27*	0.07–0.95	1.21	0.85–1.74	0.77	0.33–1.80
9 or more hours	0.78	0.44–1.39	0.63	0.35–1.11	0.75	0.28–2.06	2.37	0.53–10.58	0.80	0.38–1.66	1.26	0.31–5.08
Environment												

**Soda available in the home (ref=never)**												
Always or almost always	13.93***	8.92–21.74	12.67***	7.54–21.27	1.08	0.46–2.52	2.90	0.42–20.02	0.80	0.45–1.42	1.12	0.32–3.86
Sometimes or seldom	4.16***	2.85–6.07	3.76***	2.40–5.89	1.24	0.58–2.64	4.39	0.88–21.84	1.29	0.80–2.08	1.05	0.37–2.95
**Artificially–sweetened soda available in the home (ref=never)**												
Always or almost always	0.52**	0.34–0.78	0.44**	0.26–0.75	1.23	0.60–2.52	0.58	0.09–3.68	0.97	0.58–1.61	4.21*	1.29–13.82
Sometimes or seldom	1.37*	1.02–1.83	0.70*	0.49–0.99	1.12	0.65–1.91	0.40	0.11–1.44	0.88	0.60–1.28	2.05	0.78–5.34
**Fruit juice available in the home (ref=never)**												
Always or almost always	1.39	0.84–2.30	2.11*	1.19–3.74	3.04	0.89–10.36	0.12*	0.02–0.80	1.31	0.66–2.59	1.79	0.48–6.65
Sometimes or seldom	1.07	0.68–1.68	1.32	0.80–2.19	2.17	0.68–6.96	0.27	0.06–1.21	1.04	0.56–1.93	0.88	0.28–2.73

Note: ref indicates reference category. Variables included in each model but not statistically significant at p<0.05 for any drink type: Parental status; BMI perception; Shiftwork and Ever been diagnosed with arthritis or gout or lung condition such as Asthma or COPD. * $p < 0.05$; ** $p < 0.01$; *** $p < 0.001$.

Table S4: Hierarchical logistic regression analyses examining demographic characteristics (block 1), other drink types (block 2), and current health, behavior and environment variables (block 3) as predictors of fruit juice, artificially–sweetened soda and pre–packaged bottled water consumption (Daily/weekly versus monthly or less) for males and females separately.

	Fruit juices (any type)				Artificially–sweetened soda				Pre–packaged bottled water			
	Male		Female		Male		Female		Male		Female	
	OR	95% CI	OR	95% CI	OR	95% CI	OR	95% CI	OR	95% CI	OR	95% CI
**(Prevalence of daily/weekly consumption)**	**(46.9%)**		**(31.0%)**		**(19.1%)**		**(17.0%)**		**(39.7%)**		**(35.2%)**	
Block 1												
**Age (ref= 61 years and over)**												
18–30 years	1.30	0.97–1.74	1.82**	1.27–2.62	1.08	0.74–1.57	1.56	0.96–2.54	2.04***	1.51–2.77	2.27***	1.57–3.27
31–45 years	0.93	0.66–1.32	1.15	0.77–1.72	1.13	0.73–1.76	2.20**	1.34–3.60	1.31	0.92–1.88	1.85**	1.25–2.76
46–60 years	0.94	0.68–1.32	1.02	0.73–1.42	1.19	0.78–1.80	1.83**	1.20–2.79	0.86	0.61–1.23	2.07***	1.49–2.89
**Disadvantage (ref=least disadvantage (deciles 8–10))**												
Most disadvantage (Deciles 1–3)	1.11	0.83–1.49	1.30	0.95–1.79	1.25	0.87–1.81	1.30	0.88–1.92	1.16	0.86–1.57	1.10	0.81–1.50
Mid disadvantage (Decile 4–7)	1.02	0.80–1.29	1.09	0.85–1.41	1.25	0.93–1.69	1.24	0.91–1.69	0.98	0.76–1.25	0.98	0.77–1.25
**Education (ref=completed bachelor degree or higher)**												
Secondary school or less	0.99	0.75–1.29	0.87	0.65–1.16	1.20	0.86–1.67	0.68*	0.47–0.98	1.09	0.82–1.45	1.58**	1.19–2.09
Some tertiary or finished vocational	0.92	0.72–1.17	1.01	0.78–1.32	0.76	0.56–1.04	0.81	0.59–1.12	1.20	0.93–1.55	1.49**	1.15–1.92
**Employment (ref=currently working)**												
Not currently working	1.00	0.78–1.28	1.45**	1.13–1.86	0.64**	0.47–0.88	1.03	0.76–1.39	0.54***	0.42–0.7	0.78*	0.61–0.99
**Remoteness (ref=major cities)**												
Inner regional	0.84	0.64–1.11	0.87	0.65–1.16	0.71	0.49–1.01	1.09	0.78–1.54	1.05	0.79–1.40	0.72*	0.54–0.96
Outer regional, remote, very remote	0.82	0.58–1.15	0.86	0.58–1.26	0.68	0.43–1.08	0.76	0.47–1.25	1.00	0.70–1.42	0.91	0.63–1.31
**Grocery buyer (ref=yes or shared responsibility)**												
No	0.91	0.74–1.12	1.57**	1.12–2.20	1.07	0.82–1.39	0.95	0.61–1.48	0.91	0.73–1.12	1.29	0.92–1.81
**Speak English at home (ref=yes)**												
No	2.08***	1.45–2.98	2.06**	1.35–3.13	0.43**	0.25–0.73	0.63	0.35–1.14	1.73**	1.22–2.47	1.77**	1.17–2.68
Block 2 (Controlling for block 1)												
**Daily or weekly consumption of... (ref=no)**												
Soda	2.20***	1.76–2.75	2.56***	1.96–3.36	1.79***	1.36–2.35	2.05***	1.49–2.82	1.17	0.93–1.47	1.49**	1.14–1.95
Energy drinks	1.49	0.95–2.35	1.14	0.51–2.58	0.78	0.45–1.35	0.31	0.08–1.20	1.16	0.75–1.80	0.66	0.29–1.50

Sports drinks	1.56**	1.14–2.14	1.24	0.61–2.52	1.09	0.75–1.58	2.57**	1.28–5.17	0.97	0.71–1.32	1.33	0.68–2.61
Fruit juices (any type)					1.08	0.83–1.40	0.96	0.71–1.29	1.05	0.85–1.30	1.58***	1.25–1.98
Artificially–sweetened soda	1.09	0.84–1.41	0.95	0.71–1.28					1.37*	1.06–1.78	1.30	0.99–1.72
Pre–packaged bottled water	1.04	0.84–1.29	1.58***	1.25–1.99	1.37*	1.06–1.78	1.30	0.99–1.72				
Block 3 (Controlling for blocks 1 & 2)												
Current health												
**BMI (ref=Underweight/healthy–up to 25)**												
Overweight (25.1–29.9)	0.86	0.63–1.18	0.93	0.64–1.34	1.25	0.84–1.88	1.74*	1.08–2.79	1.53**	1.17–2.02	0.99	0.72–1.35
Obese (30–75)	0.79	0.51–1.23	1.08	0.68–1.70	1.77*	1.06–2.97	1.48	0.84–2.61	1.54*	1.06–2.23	1.29	0.88–1.89
Don’t know	0.54	0.20–1.41	0.74	0.39–1.40	1.33	0.48–3.66	1.59	0.72–3.50	1.39	0.65–2.98	0.91	0.52–1.58
**Ever had any of the following conditions (ref=No)**												
Type 2 Diabetes	1.17	0.66–2.09	0.86	0.48–1.56	2.00*	1.02–3.93	1.57	0.81–3.03	1.17	0.71–1.94	2.00**	1.26–3.18
Heart Disease	0.87	0.52–1.47	1.37	0.74–2.54	0.86	0.45–1.64	1.64	0.73–3.70	0.61*	0.37–0.99	0.86	0.47–1.56
Depression	1.14	0.80–1.61	0.65*	0.46–0.91	0.73	0.47–1.13	1.03	0.69–1.56	1.03	0.76–1.39	0.81	0.61–1.08
Behaviour												
**Takeaway food past 7 days (ref=not at all)**												
About 1–2 times	1.75***	1.30–2.36	1.02	0.77–1.35	1.00	0.68–1.47	1.39	0.98–1.97	0.98	0.76–1.26	1.24	0.98–1.56
About 3 or more times	1.82**	1.18–2.82	1.89*	1.05–3.41	1.36	0.80–2.31	1.75	0.86–3.55	1.29	0.89–1.87	1.85*	1.12–3.05
**Alcohol consumption (ref=no)**												
Consume alcohol daily or weekly	1.18	0.90–1.56	1.05	0.79–1.41	1.02	0.72–1.44	0.95	0.66–1.37	1.12	0.89–1.42	1.16	0.91–1.47
Consume spirits daily or weekly	1.10	0.73–1.64	1.15	0.69–1.91	1.48	0.91–2.4	1.62	0.89–2.94	2.17***	1.52–3.09	1.05	0.69–1.59
**Physical activity past 7 days (ref=none)**												
Some days (1 to 6 days)	1.37	0.81–2.32	1.03	0.63–1.70	0.89	0.48–1.62	1.07	0.58–1.96	0.66	0.43–1.03	0.89	0.59–1.35
Every day (7 days)	1.00	0.58–1.73	1.32	0.79–2.21	0.61	0.32–1.16	0.85	0.44–1.61	0.80	0.51–1.26	0.87	0.57–1.34
**Sleep (ref=6 hours or less)**												
7 to 8 hours	0.97	0.74–1.28	0.82	0.61–1.11	0.88	0.62–1.23	1.21	0.83–1.76	1.05	0.83–1.33	1.00	0.78–1.29
9 or more hours	0.67	0.38–1.16	1.85*	1.11–3.10	1.05	0.52–2.11	1.19	0.60–2.35	0.86	0.53–1.41	1.19	0.77–1.84
Environment												
**Soda available in the home (ref=never)**												
Always or almost always	0.78	0.51–1.20	0.64	0.40–1.00	0.18***	0.11–0.32	0.24***	0.13–0.42	1.11	0.77–1.6	1.16	0.80–1.70

Sometimes or seldom	1.08	0.78–1.50	0.86	0.62–1.18	0.40***	0.26–0.62	0.47***	0.31–0.70	1.17	0.88–1.55	1.12	0.86–1.47
**Artificially–sweetened soda available in the home (ref=never)**												
Always or almost always	0.61*	0.40–0.92	0.76	0.48–1.21	81.18***	45.60–144.54	71.77***	41.92–122.87	0.80	0.56–1.15	1.45	0.99–2.12
Sometimes or seldom	0.72*	0.54–0.97	1.07	0.79–1.45	13.60***	8.05–23.00	10.63***	6.60–17.11	1.10	0.85–1.42	1.28	0.99–1.66
**Fruit juice available in the home (ref=never)**												
Always or almost always	84.35***	42.44–167.64	93.28***	44.32–196.32	0.47*	0.26–0.86	0.58	0.33–1.02	1.09	0.72–1.65	1.37	0.93–2.01
Sometimes or seldom	8.84***	4.55–17.18	12.38***	6.01–25.50	0.59	0.34–1.01	0.57*	0.36–0.89	1.09	0.76–1.57	1.15	0.84–1.57

Note: ref indicates reference category. Variables included in each model but not statistically significant at p<0.05 for any drink type: Parental status; BMI perception; Shiftwork and Ever been diagnosed with arthritis or gout or lung condition such as Asthma or COPD. * $p < 0.05$; ** $p < 0.01$; *** $p < 0.001$.
